# Novel evidence for within-species leaf economics spectrum at multiple spatial scales

**DOI:** 10.3389/fpls.2015.00901

**Published:** 2015-10-26

**Authors:** Yu-Kun Hu, Xu Pan, Guo-Fang Liu, Wen-Bing Li, Wen-Hong Dai, Shuang-Li Tang, Ya-Lin Zhang, Tao Xiao, Ling-Yun Chen, Wei Xiong, Meng-Yao Zhou, Yao-Bin Song, Ming Dong

**Affiliations:** ^1^Key Laboratory of Hangzhou City for Ecosystem Protection and Restoration, College of Life and Environmental Sciences, Hangzhou Normal UniversityHangzhou, China; ^2^State Key Laboratory of Vegetation and Environmental Change, Institute of Botany, Chinese Academy of SciencesBeijing, China; ^3^Institute of Wetland Research, Chinese Academy of ForestryBeijing, China

**Keywords:** intraspecific variation, leaf economics spectrum (LES), leaf economic traits, spatial scales, trade-offs, trait relationships, wetland plant

## Abstract

Leaf economics spectrum (LES), characterizing covariation among a suite of leaf traits relevant to carbon and nutrient economics, has been examined largely among species but hardly within species. In addition, very little attempt has been made to examine whether the existence of LES depends on spatial scales. To address these questions, we quantified the variation and covariation of four leaf economic traits (specific leaf area, leaf dry matter content, leaf nitrogen and phosphorus contents) in a cosmopolitan wetland species (*Phragmites australis*) at three spatial (inter-regional, regional, and site) scales across most of the species range in China. The species expressed large intraspecific variation in the leaf economic traits at all of the three spatial scales. It also showed strong covariation among the four leaf economic traits across the species range. The coordination among leaf economic traits resulted in LES at all three scales and the environmental variables determining variation in leaf economic traits were different among the spatial scales. Our results provide novel evidence for within-species LES at multiple spatial scales, indicating that resource trade-off could also constrain intraspecific trait variation mainly driven by climatic and/or edaphic differences.

## Introduction

Understanding species’ trait variation and covariation is critical to explain species’ strategies in response to environmental gradients and ecosystem functioning (Westoby et al., 2002; Garnier et al., 2004; Lavorel and Grigulis, 2012). Some important trade-offs, underpinning ecological strategies, have been found among species, e.g., C-S-R triangle (Grime, 1979), leaf-height-seed (LHS) strategy scheme (Westoby, 1998) and leaf economics spectrum (LES; Wright et al., 2004). LES, describing the covariation among leaf economic traits related to resource acquisition and conservation (Wright et al., 2004; Reich, 2014), provides a paradigm or framework for checking species strategies shaped by evolutionary history (Donovan et al., 2011; Reich, 2014). Recently, LES was found to be modulated by climate and biogeographic factors (Wright et al., 2005; Heberling and Fridley, 2012). Moreover, some researchers attempted to extend LES to ‘wood economics spectrum’ (Chave et al., 2009) and ‘plant economics spectrum’ (Freschet et al., 2010, 2012). In addition, researchers have done much work on the origin of LES (Shipley et al., 2006; Blonder et al., 2011; Vasseur et al., 2012).

Although much progress has been made in LES among species, few studies investigated the application of LES within species (but see Blonder et al., 2013; Niinemets, 2015). The main reasons may be related to the expected lower variation and much less concern about variation within species than that among species (Fajardo and Piper, 2011). However, with increasing concern about intraspecific variation, large variability of functional traits was found within species (Albert et al., 2010; Messier et al., 2010), especially for widespread species because of their genetic and plastic variation (Darwin, 1859; Sides et al., 2014). In addition, recent studies in a number of widespread plant species showed that some trait-based trade-offs within species were either consistent (Fajardo and Piper, 2011; Richardson et al., 2013) or inconsistent (De Frenne et al., 2011; Hajek et al., 2013) with that among species. Therefore, there is need to examine whether LES, a trade-off largely reported among species, exists within species. Among the few studies about within-species LES, Blonder et al. (2013) studied the LES within clones of the tree *Populus tremuloides*, while Niinemets (2015) identified the LES in a shrub, *Quercus ilex*, across the Mediterranean region. Jackson et al. (2013) found that within-species LES occurred in 11 of 16 species across a temperate rain forest. Although these studies provided some evidence for within-species LES, the results were not consistent for all species investigated. Apparently, within-species LES was only examined in forest plant species.

Trait variation exists at all temporal, spatial, and organizational scales: individual, population, species, community, local, and regional (Albert et al., 2010; Messier et al., 2010). It has been known that different spatial scales associated with differences in climatic and/or edaphic conditions can be great drivers of variation and covariation in leaf economic traits (Liu et al., 2010; Messier et al., 2010). Therefore, assessing LES across different spatial scales can provide insight into the causes of LES (Blonder et al., 2013). Among-species LES has been extensively studied and identified at local, regional, and global scales (Díaz et al., 2004; Wright et al., 2004; Freschet et al., 2010; Jackson et al., 2013). As for within-species LES, previous studies were conducted at single organizational or spatial scale like within-clone (Blonder et al., 2013) or regional scale (Jackson et al., 2013; Niinemets, 2015). Therefore, it is still unclear how within-species LES varies across different spatial scales, or whether it is scale-dependent.

*Phragmites australis*, a perennial grass of Poaceae, is a cosmopolitan wetland species. As a dominant species in many wetland ecosystems, *P. australis* provides a number of important ecosystem services, e.g., water purification, paper production, and energy production (Thevs et al., 2007). It occurs along wide climatic gradients, ranging from temperate to tropic regions and from arid to humid regions in China as well as in the world (Editorial Committee of Wetland Vegetation in China, 1999). Large variation of traits in *P. australis* was found at regional scales due to the wide environmental range and genetic variation (Clevering et al., 2001; Lambertini et al., 2008). Therefore, it is an ideal plant species for the study of intraspecific variation and covariation in traits across different spatial scales. A few studies explored the trait variation in *P. australis* along large-scale environmental gradients (Clevering et al., 2001; Drapikowska and Krzakowa, 2009; Li et al., 2014). But none of them examined the covariation in leaf economic traits of *P. australis* along wide climatic gradients or at multiple spatial scales.

In this study, we aimed to investigate the variation and covariation in leaf economic traits of *P. australis* across different spatial scales (inter-regional, regional, and site) in China. Thus, we carried out a 3-years field investigation on 16 natural wetlands covering most of the geographic range of *P. australis* across China, and quantified four leaf economic traits of *P. australis*: specific leaf area (SLA), leaf dry matter content (LDMC), leaf nitrogen (N), and leaf phosphorus (P) concentration. Specifically, we attempted to answer the following questions: (1) How do leaf economic traits vary across different spatial scales? (2) Do the leaf economic traits vary to form within-species LES across the species range? (3) Does the existence of within-species LES depend on spatial scales? (4) How do climate and soil variables influence leaf economic traits across different spatial scales?

## Materials and Methods

### Study Sites and Sampling Methods

**FIGURE 1:**
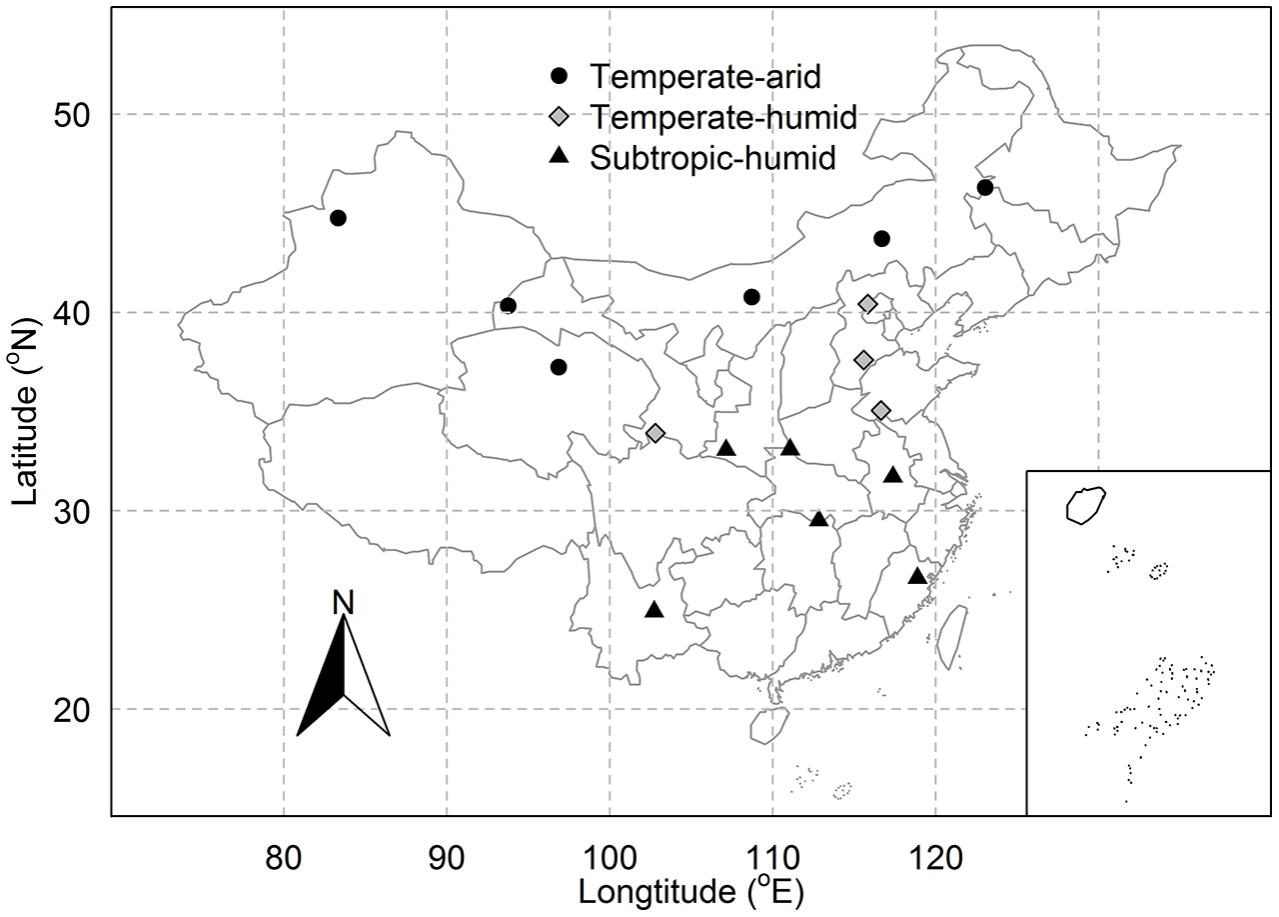
**Distribution of sampling sites for *Phragmites australis* across China**. Different types of symbols represent sites of different regions.

*Pragmites australis* is distributed over large areas of China: from temperate to tropic regions and from arid to humid regions (Editorial Committee of Wetland Vegetation in China, 1999). Previous studies have found a large extent of trait variation in *P. australis* due to genetic and plastic variation across China (Editorial Committee of Wetland Vegetation in China, 1999; Zhang et al., 2003; An et al., 2012). To investigate the intraspecific variation and covariation in *P. australis*, we chose 16 sites in natural wetlands which covered most of the distribution range of *P. australis* in China (**Figure 1**) and which constituted broad climatic gradients with mean annual temperature (MAT) ranging from 1.6 to 17.4°C and mean annual precipitation (MAP) from 40 to 1702 mm (**Table 1**).

**Table 1:** Summary of leaf economic traits of *Phragmites australis* and environmental variables based on plot mean values.

Variables	*N*	Mean	*SD*	CV (%)	Minimum	Maximum
**Leaf economic trait**						
SLA (mm^2^ mg^-1^)	53	13.6	3.54	25.9	7.6	24.7
LDMC (mg g^-1^)	53	394	51.9	13.2	287	526
Leaf N (mg g^-1^)	54	25.9	7.42	28.6	10.9	45.4
Leaf P (mg g^-1^)	54	1.5	0.55	36.7	0.7	3.5
**Environmental variable**						
MAT (°C)	55	10.6	4.66	43.9	1.6	17.4
MAP (mm)	55	543	433	79.9	40	1702
Soil pH	52	8.67	0.70	8.1	6.79	10.61
Soil EC (ms cm^-1^)	53	5.59	9.82	175.6	0.03	42.12
Soil N (mg g^-1^)	52	1.9	1.55	83.8	0.2	7.7
Soil P (mg g^-1^)	51	0.7	0.22	31.5	0.3	1.3
Soil C/N	52	18.9	11.75	62.3	7.4	72.1
Soil organic C (mg g^-1^)	52	18.2	16.20	89.1	2.2	75.4
Soil available N (mg kg^-1^)	52	119	99.9	84.1	12	459
Soil available P (mg kg^-1^)	53	19.2	17.33	90.3	1.5	79.0

*SLA, specific leaf area; LDMC, leaf dry matter content. Full names of other variables were given in Section “Materials and Methods.”*

Three nested spatial scales were identified in this study: inter-regional, regional, and site. At the inter-regional scale, all sampling sites across China were included. For the regional scale, the 16 study sites were distributed in three regions based on temperature and precipitation: temperate-arid, temperate-humid, and subtropic-humid region (Editorial Board of Physical Geography in China Chinese Academy of Sciences, 1985; **Figure 1**). As a result, there were six sites in temperate-arid, four in temperate-humid and six in subtropic-humid region. Within each site, 1–6 plots each about 20 m × 20 m were chosen with a distance of 10–30 km for two adjacent plots. In total, 55 plots across China were chosen. In each plot, several (about 3–10) adult individuals of *P. australis* without obvious symptoms of pathogen or herbivore attack were randomly selected. Then, we picked some mature and fully expanded sun leaves (about 1–5 based on the size of leaves) from each individual. Leaves of *P. australis* from all individuals at each plot were pooled together, divided into three batches and stored in sealed plastic bags within 8 h before being determined for SLA and LDMC (Pérez-Harguindeguy et al., 2013). Leaf sampling and morphological measurements were conducted during the growing seasons from 2012 to 2014. Since sampling was carried out at three adjacent years and wetlands were insensitive to precipitation fluctuation, main climate variation between years, the inter-annual variation in traits was not considered in this study.

### Leaf Economic Traits

We selected SLA, LDMC, leaf N and P, which are key traits in LES (Wright et al., 2004; Freschet et al., 2010). Specifically, SLA represents the light intercept capability with per unit of dry-mass investment, and is related to photosynthetic capacity (Pérez-Harguindeguy et al., 2013). LDMC reflects the dry-mass investment in leaves (Freschet et al., 2010). Leaf N, mostly in proteins, is closely related to the mass-based maximum photosynthetic rate, and leaf P, high in nucleic acid, lipid membranes, and bioenergetic molecules such as adenosine triphosphate, is important in metabolic process (Wright et al., 2004). Thus, resource acquisition strategist is generally characterized by high SLA, leaf N and P while resource conservation strategist by high LDMC.

All leaf samples were determined for the four leaf economic traits. The same leaf samples were used during the whole process of measurements. Firstly, one sample from each leaf batch was immersed in water overnight, blotted up water and measured for water-saturated weight. Secondly, the same leaf samples were scanned with a photo scanner (CanoScan LiDE210; Canon, Japan) and weighted after oven-dried at 70°C for 72 h. Thirdly, leaf area of each sample was accessed from scanned photo with ImageJ (http://imagej.nih.gov/ij/). Then, SLA (mm^2^ mg^-1^) of each sample was calculated as the ratio of sample leaf area to oven-dry weight. LDMC (mg g^-1^) was determined by the ratio of leaf oven-dry weight to water-saturated weight. Leaf N (mg g^-1^) was determined using an elemental analyzer (vario MICRO cube; Elemental, Germany), while leaf P (mg g^-1^) was determined using ascorbic acid colorimetric method after H_2_SO_4_ digestion as described by Bao (2005).

### Environmental Variables

Mean annual temperature and MAP were accessed from published studies which were carried out at the same sites. For each plot, three replicates of soil samples from 0 to 15 cm depth were randomly excavated. Soil samples were brought into the laboratory, air dried and passed through a 1 mm-sieve before measurement. For soil pH, 5 g subsample of each soil sample was shaken with 25 mL demineralized water in Eppendorf tube for 30 min at 250 rpm and measured for pH after standing for 30 min. The solution from pH measurement was then used to measure soil electrical conductivity (soil EC, ms cm^-1^): the solution was centrifuged at 5000 rpm for 5 min, and the supernatant solution was measured for EC. Soil available N (mg kg^-1^) was measured with the alkaline hydrolysis diffusion method, while soil available P (mg kg^-1^) was determined by Olsen method (Bao, 2005).

For soil total carbon, nitrogen, and phosphorus content, a small amount of each soil sample which passed through a 0.15 mm-sieve was used. Soil total carbon (soil C, mg g^-1^), nitrogen (soil N, mg g^-1^), and phosphorus contents (soil P, mg g^-1^) were determined following the same methods as for leaf N and P (see above). In addition, we measured soil organic C (mg g^-1^) by subtracting soil inorganic content from soil total carbon, which were measured using a TOC analyzer (SSM 5000A; Schimadzu, Japan). All air-dry soil samples were oven-dried at 105°C for 6 h and measured for water content, and soil nutrient content of samples were expressed on an oven-dry mass basis.

### Statistical Analyses

We used plot-level means of traits for all analyses. Descriptive statistics including mean, standard deviation (SD) and coefficient of variation (CV) were calculated for each trait. To quantify the extent of variation across three spatial scales, we used nested ANOVA with restricted maximum likelihood (REML) method to estimate the variance component across scales (Messier et al., 2010). Because our aim was only to calculate the variance of traits at each scale, the data were not transformed before nested ANOVA (Quinn and Keough, 2002). Instead, SLA, leaf P, MAP, soil N, soil P, soil C/N, soil organic C, available N, available P and EC were log_10_-transformed before the following data analysis to meet the assumption of approximate normality and residuals homogeneity, while data sets of LDMC, leaf N, MAT and soil pH were not transformed because they met approximately normal distribution. Simple linear regression with ordinary least-squares method was used to explore bivariate trait-trait relationships at three spatial scales (inter-regional, regional, and site). Notably, at site scale, ordinary least-squares regression was only performed for sites with more than three plots, i.e., 12 of total 16 sites. Pearson’s correlation analysis was carried out to quantify pairwise relationship between leaf traits and environmental variables at inter-regional and regional scale other than at site scale owing to limited data. Statistical significance of each correlation was assessed after Holm–Bonferroni correction for multiple comparisons. Principal component analysis (PCA) was also used to explore the correlations among traits and account for the majority of variation at inter-regional and regional scales. All analyses were performed in R 3.1.0 (R Core Team, 2014).

## Results

### Trait Variation in *P. australis* across Different Spatial Scales

**FIGURE 2:**
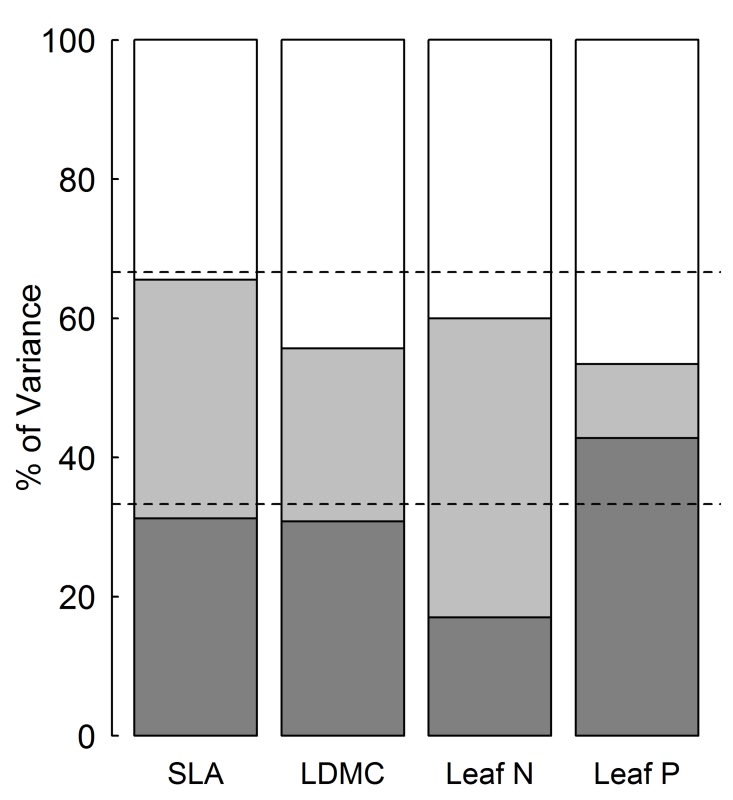
**Variance partitioning for leaf economic traits across three spatial scales**. SLA, specific leaf area; LDMC, leaf dry matter content; leaf N, leaf nitrogen content; leaf P, leaf phosphorus content. 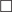
Plot, 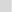
Site, 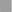
Region. The 33.3 and 66.6% thresholds are given by dash lines.

The intraspecific variation in four leaf economic traits of *P. australis* was different (**Table 1**). LDMC varied the least, ranging from 287 to 526 mg g^-1^ (CV = 13.2%), while leaf P varied the most, from 0.7 to 3.5 mg g^-1^ (CV = 36.7%). The extent of variation in SLA and leaf N was intermediate with 25.9–28.6% of CV. Moreover, variance partitioning in nested ANOVA showed that variability in the leaf economic traits was different across spatial scales (**Figure 2**). Variability between plots (within sites) accounted for the largest proportion of total variance in SLA, LDMC, and leaf P (34.5, 44.3, and 46.6%, respectively) and the second largest in leaf N (40.0%). Variability between sites differed across traits, 10% of total variance for leaf P and 25–40% for other traits (**Figure 2**). The smallest variance in leaf N (17.0%) was due to difference between regions, which caused the largest variability in leaf P (42.8%). Variance components of SLA and LDMC were 31.3 and 30.8% of total variance at regional scale.

### Intraspecific Trait Covariation across Different Scales

**FIGURE 3:**
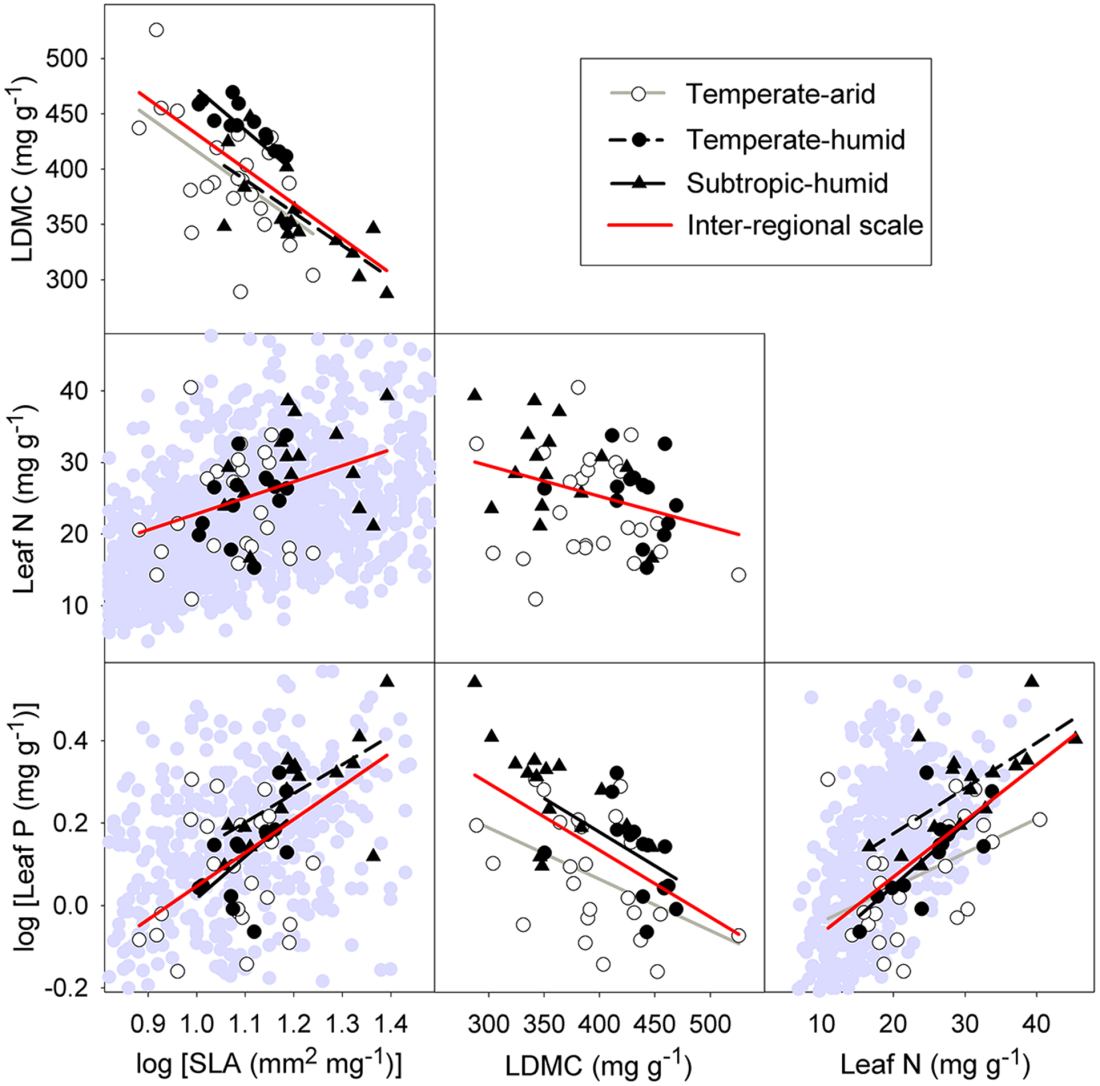
**Intraspecific relationships among four economic traits at inter-regional and regional scale**. Abbreviation for traits can be seen in **Figure 2**. Lines are plotted for relationships with *p* < 0.05. Symbols in the background are Glopnet data (Wright et al., 2004). The range is truncated to the trait ranges of *P. australis*.

At inter-regional scale, strong relationships were found among leaf economic traits of *P. australis* (**Figure 3**). Specifically, SLA was positively correlated with leaf N and P (*r^2^* = 0.12, *p* = 0.012; *r^2^* = 0.32, *p* < 0.001), but was negatively correlated with LDMC (*r^2^* = 0.42, *p* < 0.001). LDMC was significantly negatively correlated with leaf N and P (*r^2^* = 0.10, *p* = 0.021; *r^2^* = 0.30, *p* < 0.001), while leaf P was positively correlated with leaf N (*r^2^* = 0.42, *p* < 0.001).

At regional scale, significant correlations between SLA and LDMC, between LDMC and leaf P and between leaf N and P remained for three different regions: temperate-arid, temperate-humid, and subtropic-humid regions (**Figure 3**). There were weak relationships between leaf structure traits and leaf N at all three regions (**Figure 3**). Significant correlation between SLA and leaf P was only detected at the temperate-humid and subtropic-humid regions (**Figure 3**). At site scale, significant correlations among leaf economic traits were detected only in a small number of sites which are distributed in different regions (**Table 2**; Supplementary Figure S1).

**Table 2:** Summary of intraspecific relationships among four leaf economic traits of *P. australis* at site scale.

	Number of sites (*n* = 16)			Direction	Region
	*p* < 0.05	*p* < 0.10	*p* < 0.15		
LDMC-SLA	3	4	5	-	ta, th, sh
Leaf N-SLA	1	1	2	+	th
Leaf P-SLA	0	2	2	+	th, sh
Leaf N-LDMC	1	1	4	-	sh
Leaf P-LDMC	1	2	4	-	th, sh
Leaf P-leaf N	0	3	5	+	th, sh

*SLA, specific leaf area; LDMC, leaf dry matter content. Values in the first three columns is number of sites within which trait relationships are significant at *p* < 0.05, *p* < 0.10, and *p* < 0.15, respectively. Direction of trait relationships: +, positive; -, negative. Significant trait relationships (*p* < 0.10) in three regions: ta, temperate-arid; th, temperate-humid; sh, subtropic-humid*.

### Environmental Correlates of Leaf Economic Traits across Different Spatial Scales

The correlations between leaf economic traits of *P. australis* and environmental variables (climate and soil factors) were different across different spatial scales (**Table 3**). Specifically, at inter-regional scale, SLA and leaf P were all strongly related to MAT, MAP, soil pH, soil EC and soil C/N, except that the correlations between SLA, MAT, and soil pH were not significant (*p* > 0.05, **Table 3**). Leaf N was significantly related to MAP and soil pH (*p* < 0.05, **Table 3**), and LDMC decreased with increasing soil P and available P (**Table 3**). At regional scale, in temperate-arid region, SLA was significantly correlated with soil EC, while leaf N was significantly related to MAT, and leaf P related to MAP (**Table 3**). In contrast, LDMC was only significantly positively correlated with MAT in temperate-humid region (**Table 3**). In subtropic-humid region, SLA and leaf P were positively related to soil EC, and SLA decreased with increasing MAT (**Table 3**).

**Table 3:** Pearson’s correlation between leaf economic traits of *P. australis*, climate and soil variables at the inter-regional and regional (temperate-arid, temperate-humid, and subtropical-humid) scales.

Scale	Variables	SLA	LDMC	Leaf N	Leaf P
Inter-regional	MAT	0.28	-0.04	0.20	**0.38**
	MAP	**0.49**	-0.16	**0.49**	**0.60**
	Soil pH	-0.30	0.12	**-0.51**	**-0.42**
	Soil EC	**-0.52**	0.06	-0.22	**-0.39**
	Soil N	0.11	0.05	0.12	-0.09
	Soil P	0.33	**-0.54**	0.01	0.21
	Soil C/N	**-0.54**	0.31	-0.36	**-0.43**
	Soil organic C	0.01	0.12	0.03	-0.17
	Soil available N	0.10	0.04	0.18	-0.02
	Soil available P	0.14	**-0.45**	0.32	0.17
Temperate-arid region	MAT	-0.41	0.17	**-0.57**	-0.43


	MAP	0.44	-0.27	0.55	**0.56**
	Soil pH	-0.06	-0.08	-0.25	-0.03
	Soil EC	**-0.64**	0.26	0.01	-0.08
	Soil N	0.44	0.06	0.13	-0.17
	Soil P	0.45	-0.38	-0.20	0.26
	Soil C/N	-0.47	0.26	-0.26	-0.10
	Soil organic C	0.48	0.02	0.03	-0.20
	Soil available N	0.41	0.08	0.08	-0.16
	Soil available P	0.31	-0.37	0.30	-0.03
Temperate-humid region	MAT	-0.41	**0.80**	0.06	0.03


	MAP	-0.16	-0.05	-0.02	-0.01
	Soil pH	-0.41	0.49	-0.49	-0.28
	Soil EC	0.00	-0.09	0.45	0.28
	Soil N	-0.23	0.39	0.00	-0.17
	Soil P	0.47	-0.50	0.27	0.15
	Soil C/N	0.20	0.03	0.29	0.49
	Soil organic C	-0.26	0.36	0.08	-0.14
	Soil available N	-0.25	0.22	0.22	0.07
	Soil available P	0.52	-0.70	0.56	0.46
Subtropic-humid region	MAT	**-0.75**	0.32	0.15	-0.37


	MAP	-0.61	0.12	0.13	-0.34
	Soil pH	0.62	-0.43	-0.43	0.08
	Soil EC	**0.73**	-0.61	0.55	**0.76**
	Soil N	-0.03	-0.14	0.41	0.47
	Soil P	0.50	-0.54	0.06	0.41
	Soil C/N	0.36	-0.27	0.23	0.22
	Soil organic C	-0.16	0.00	0.36	0.35
	Soil available N	-0.24	-0.03	0.51	0.33
	Soil available P	-0.06	0.02	0.46	0.45

*Values are Pearson’s correlation coefficients. Values in bold indicate significance at *p* < 0.05 after Holm-Bonferroni correction for the number of tests. SLA, leaf P, MAP, soil N, soil P, soil C/N, soil organic C, soil available N, soil available P, soil EC were log_*10*_-transformed, other variables were not. Full name of variables were given in “*Materials and Methods*.”*

## Discussion

### Multi-scale Intraspecific Variation in Leaf Economic Traits

We found a substantial amount of variation in leaf economic traits for the cosmopolitan wetland species *P. australis* across most of its geographic range in China (**Table 1**, Supplementary Figure S2). Although intraspecific trait variation was less than interspecific variation across China and the globe, it covered a large proportion of interspecific variation at scales either the same as or much larger than that in this study (Supplementary Figure S2). The large trait variation in *P. australis* was consistent with results of Albert et al. (2010) and Fajardo and Piper (2011) in other species along large environmental gradients. Although the extent of trait variation may depend on species and traits, an increasing number of studies indicate the important role of intraspecific trait variation at community level (Albert et al., 2010; Messier et al., 2010; Jackson et al., 2013). These facts imply that intraspecific variation is an important component of trait variation, which should not be neglected in investigating the responses of communities and ecosystems to environmental changes (Fajardo and Piper, 2011; Jackson et al., 2013).

On the other hand, the distribution of variance in SLA and LDMC was relatively uniform among three spatial scales (**Figure 2**). Since different spatial scales are associated with differences in ecological processes, such as genetic variation, edaphic and climatic conditions (Messier et al., 2010), our results indicate that processes driving variation at different spatial scales were of similar importance for the leaf structural traits considered. In addition, small variance for leaf P at site scale and leaf N at regional scale suggests that climatic condition at regional scale may play a critical role in driving leaf P rather than leaf N. Overall, the variation in all four leaf economic traits at site and regional scale were comparable to that at inter-regional scale (**Figure 2**). The large intraspecific trait variability at local (site) scale was consistent with previous studies within species (Albert et al., 2010; Fu et al., 2013) and among species (Wright et al., 2004). It suggests that environmental heterogeneity (mainly in terms of soil nutrients and water) at local scale plays an important role in driving leaf economic traits of species.

### Existence of Within-species LES across the Species Range

We found strong correlations among the four leaf economic traits (SLA, LDMC, leaf N and P) of *P. australis* related to resource acquisition and conservation across the species range in China (**Figure 3**; Supplementary Figure S3A), which indicates that within-species LES occurs in the widespread wetland grass across a large scale. Consistent with results from Blonder et al. (2013) in a clonal tree (*Populus tremuloides*) and Niinemets (2015) in a Mediterranean shrub (*Quercus ilex*), our results provide evidence for within-species LES. This observed LES within species was similar to LES found among species (**Figure 3**; Wright et al., 2004; Freschet et al., 2010). It suggests that the trade-off between resource acquisition and conservation, which has been extensively found among species, is likely to operate within species. Additionally, previous work showed that trait variation in within-species LES could result from environmental difference (Blonder et al., 2013), genetic variation (Vasseur et al., 2012), or both (Jackson et al., 2013; Niinemets, 2015). This study confirmed those, which meant resource trade-off could constrain intraspecific trait variation driven by genetic and environmental variation.

Although some studies, including this one, found within-species LES in different species, it is still far from a general conclusion. It is necessary to examine more species of different growth forms and in different habitats. As within-species LES exists widely, a quantitative comparison is needed to explore the potential differences between intraspecific and interspecific LES. We found some qualitative differences between LES in *P. australis* and among species, e.g., the relationship between leaf N and P (**Figure 3**; Wright et al., 2004). Considering the large extent of trait variation within species and the potential contrasts in LES between within and among species, we argue that intraspecific trait variation needs to be incorporated into worldwide LES and global dynamic vegetation models, which will provide a better understanding and prediction of global change responses (Moran et al., 2015; Niinemets, 2015).

### Within-species LES across Different Spatial Scales

The relationships between leaf structural traits (SLA and LDMC) and leaf N of *P. australis* were significant at inter-regional scale, but not at regional scale (**Figure 3**). Meanwhile, there existed some differences in trait relationships between site and regional scales, although the data at site scale was limited. These were consistent with previous studies which already provided some evidence for scale-dependency of trait relationships (Burns and Beaumont, 2009; Liu et al., 2010). Different environmental gradients and biotic factors occurring among different spatial scales were likely to influence the scale-dependency of trait relationships.

Although there were some differences in trait relationships among the three spatial scales, coordination among the leaf economic traits resulted in LES at both inter-regional and regional scales (Supplementary Figure S3). This indicates that trade-off between resource acquisition and conservation, forming within-species LES, constrains the trait variation at large scale (Niinemets, 2015). At local scale, strong correlations among leaf economic traits were found in several sites, for example, in site HS (temperate-humid region), we found significant relationships among traits except between LDMC and leaf N (Supplementary Figure S1). This showed that within-species LES occurred at a local scale, which was consistent with the study by Blonder et al. (2013) on within-clone LES within each site. It suggests that trade-off generating the LES can operate within species at local scale as well (Blonder et al., 2013). In summary, our results demonstrated that within-species LES emerged at multiple spatial scales (inter-regional, regional, and local), although there were a few differences between relationships among leaf economic traits across different scales.

### Multi-scale Effects of Climate and Soil Variables on Leaf Economic Traits

At inter-regional scale, leaf economic traits were closely correlated with several climate and soil variables. At regional scale, the few predictors of leaf economic traits were MAT, MAP, and soil EC in temperate-arid region, MAT in temperate-humid region, and MAT and soil EC in subtropic-humid region. These facts indicate that the environmental variables determining leaf economic traits were different among spatial scales, although all these climate and soil factors have been found to influence leaf economic traits (Ordoñez et al., 2009; Fujita et al., 2013; Richardson et al., 2013; Maire et al., 2015).

Contrary to our expectation, soil N, soil organic C and available N, which were closely related to each other, were weak predictors of the four leaf economic traits at inter-regional and regional scales. It may be due to the phosphorus limitation of *P. australis* across wetlands in China, supported by the fact that leaf N/P of *P. australis* in 43/55 plots was higher than 16 (Koerselman and Meuleman, 1996).

Overall, relationships between leaf economic traits and environmental variables were variable among spatial scales. It suggests that distinct environmental variation at different spatial scales shapes the different functional responses of *P. australis*. Therefore, the various environmental variations resulting from multiple spatial scales should be considered in investigating the influence of climate and soil properties on species’ leaf functional traits (Ordoñez et al., 2009; De Frenne et al., 2011).

## Conclusion

A substantial amount of variation in leaf economic traits was found for the cosmopolitan wetland species *P. australis* at three spatial scales across most of its geographic range in China, indicating that variation within species is not negligible. Leaf economic traits were coordinated to form within-species LES in the wetland species *P. australis* across China, which provide novel evidence for existence of LES within species. Our results demonstrated that within-species LES occurred at multiple spatial scales (inter-regional, regional, and site). This improved our understanding on the scale-dependent aspects of within-species LES. Finally, the environmental variables determining variation in leaf economic traits were different among spatial scales. Further studies are needed to investigate the role of scale in trait variation and covariation both within and among species.

## Conflict of Interest Statement

The authors declare that the research was conducted in the absence of any commercial or financial relationships that could be construed as a potential conflict of interest.
