# Supplementary material for: Novel evidence for within-species leaf economics spectrum at multiple spatial scales
Source: Front Plant Sci. 2015 Oct 26;6:901. doi: 10.3389/fpls.2015.00901 (PMC4620397; doi:10.3389/fpls.2015.00901)
Supplement: Supplementary file 1 [file Presentation_1.PDF]

Supplementary Material

Novel evidence for within-species leaf economics spectrum at multiple spatial scales

Yu-Kun Hu[1,2], Xu Pan[1,3], Guo-Fang Liu[2], Wen-Bing Li[1], Wen-Hong Dai[1], Shuang-Li Tang[2], Ya-Lin Zhang[2], Tao Xiao[1], Ling-Yun Chen[1], Wei Xiong[1], Meng-Yao Zhou[1], Yao-Bin Song[1,*], Ming Dong[1,2,*]

***Correspondence:**
Yao-Bin Song: ybsong@hznu.edu.cn; Ming Dong: dongming@hznu.edu.cn

1 Supplementary Figures

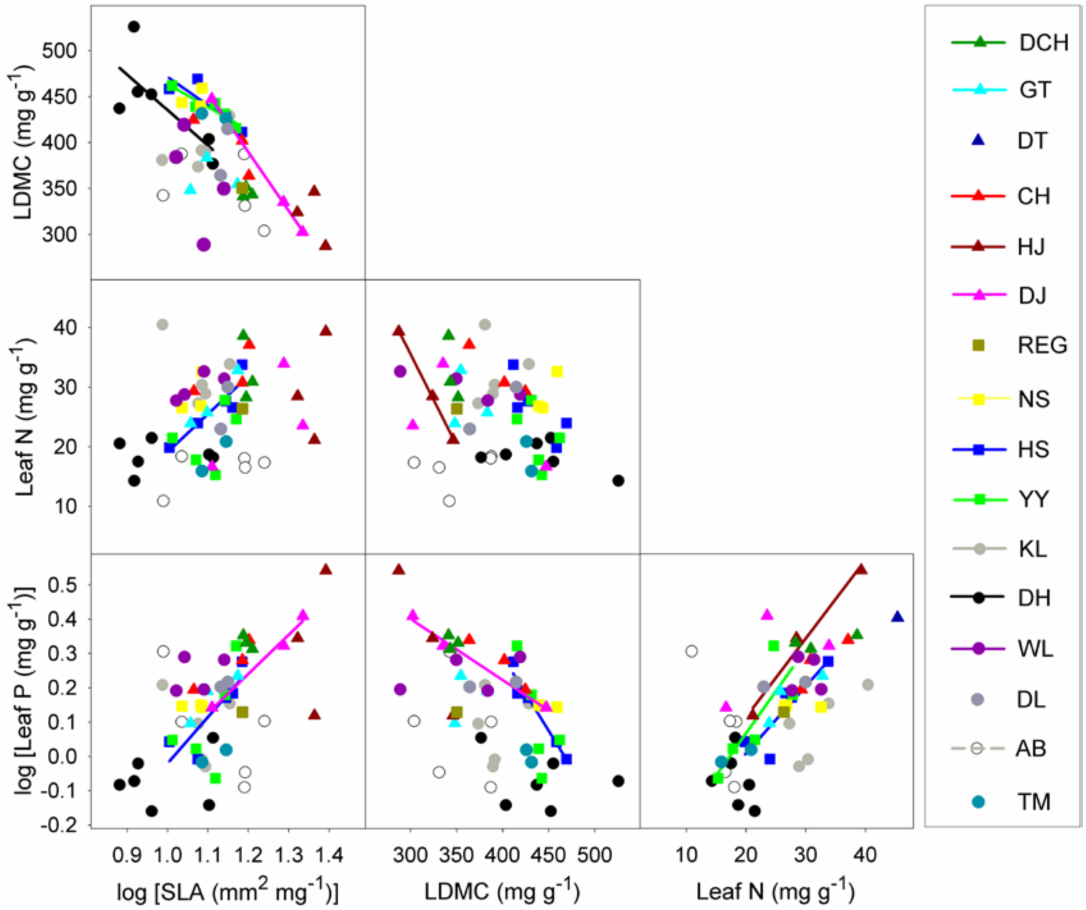

Supplementary Figure 1. Intraspecific relationships among four economic traits at site scale. SLA, specific leaf area; LDMC, leaf dry matter content. One point is one plot and points with the same color are of the same sites. Symbols with different shapes represent different regions: triangle, subtropic-humid; square, temperate-humid; circle, temperate-arid. Lines were plotted for relationships with $p < 0.10$. Regression was not performed for site DT, REG, DL and TM, each with less than three plots.

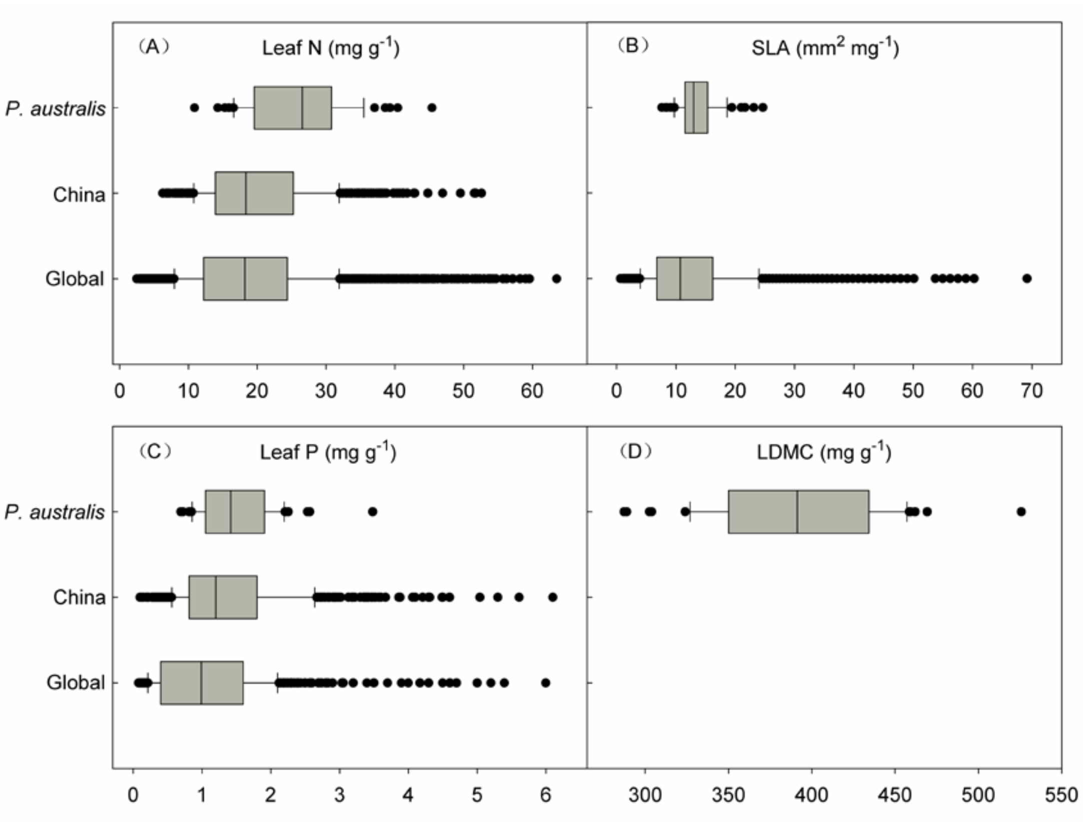

Supplementary Figure 2. Variations in leaf nitrogen (N) content, leaf phosphorus (P) content, specific leaf area (SLA) and leaf dry matter content (LDMC) of *P. australis* (this study) and among species across China (Han et al., 2005) and the globe (GLOPNET, Wright et al., 2004). SLA across China and LDMC across China and the globe were not shown due to lack of data.

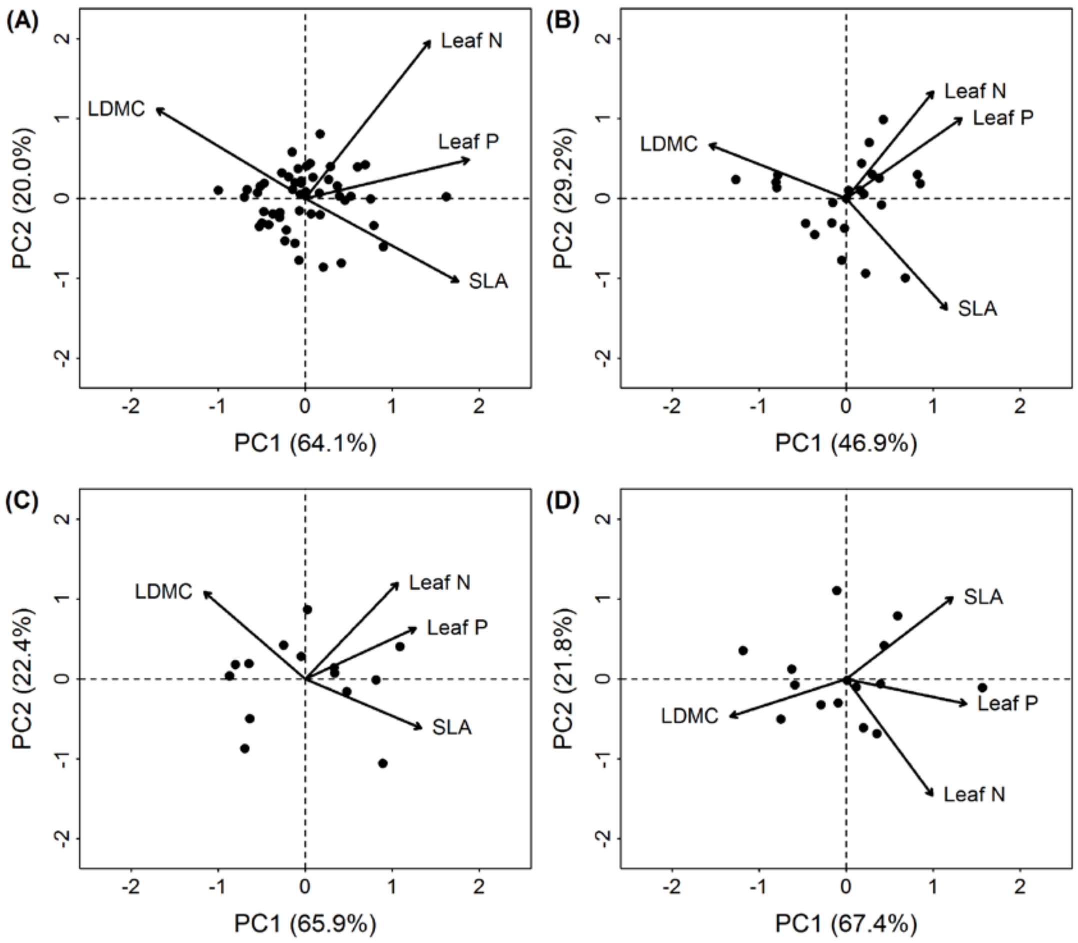

Supplementary Figure 3. PCA for the relationships among four economic traits with plots **(A)** inter-regional, **(B)** in temperate-arid region, **(C)** in temperate-humid region, **(D)** in subtropic-humid region. Abbreviation for traits can be seen in **Supplementary Figure 1**.

References

Han, W., Fang, J., Guo, D., and Zhang, Y. (2005). Leaf nitrogen and phosphorus stoichiometry across 753 terrestrial plant species in China. *New Phytol.* 168, 377-385. doi: 10.1111/j.1469-8137.2005.01530.x

Wright, I.J., Reich, P.B., Westoby, M., Ackerly, D.D., Baruch, Z., Bongers, F., ~~Cavender-Bares, J., Chapin, T., Cornelissen, J.H.C., Diemer, M., Flexas, J., Garnier, E., Groom, P.K., Gulias, J., Hikosaka, K., Lamont, B.B., Lee. T., Lee, W., Lusk, C., Midgley, J.J., Navas, M.-L., Niinemets, Ü., Oleksyn, J., Osada, N., Poorter, H., Poot, P., Prior, L., Pyankov, V.I., Roumet, C., Thomas, S.C., Tjoelker, M.G., Veneklaas, E.J., and Villar, R~~. (2004). The worldwide leaf economics spectrum. *Nature* 428, 821-827. doi:10.1038/nature02403
